# Bid Participates in Genotoxic Drug-Induced Apoptosis of HeLa Cells and Is Essential for Death Receptor Ligands' Apoptotic and Synergistic Effects

**DOI:** 10.1371/journal.pone.0002844

**Published:** 2008-07-30

**Authors:** Barbara Köhler, Sergio Anguissola, Caoimhin G. Concannon, Markus Rehm, Donat Kögel, Jochen H. M. Prehn

**Affiliations:** 1 Department of Physiology and Medical Physics, Royal College of Surgeons in Ireland, Dublin, Ireland; 2 RCSI Center for Human Proteomics, Royal College of Surgeons in Ireland, Dublin, Ireland; 3 Experimental Neurosurgery, Centre for Neurology and Neurosurgery, Johann Wolfgang Goethe University Clinics, Frankfurt/Main, Germany; Universität Heidelberg, Germany

## Abstract

**Background:**

The BH3-only protein Bid is an important component of death receptor-mediated caspase activation. Bid is cleaved by caspase-8 or -10 into t-Bid, which translocates to mitochondria and triggers the release of caspase-activating factors. Bid has also been reported to be cleaved by other proteases.

**Methodology/Principal Findings:**

To test the hypothesis that Bid is a central mediator of stress-induced apoptosis, we investigated the effects of a small molecule Bid inhibitor on stress-induced apoptosis, and generated HeLa cells deficient for Bid. Stable knockdown of *bid* lead to a pronounced resistance to Fas/CD95- and TRAIL-induced caspase activation and apoptosis, and significantly increased clonogenic survival. While Bid-deficient cells were equally sensitive to ER stress-induced apoptosis, they showed moderate, but significantly reduced levels of apoptosis, as well as increased clonogenic survival in response to the genotoxic drugs Etoposide, Oxaliplatin, and Doxorubicin. Similar effects were observed using the Bid inhibitor BI6C9. Interestingly, Bid-deficient cells were dramatically protected from apoptosis when subtoxic concentrations of ER stressors, Etoposide or Oxaliplatin were combined with subtoxic TRAIL concentrations.

**Conclusions/Significance:**

Our data demonstrate that Bid is central for death receptor-induced cell death and participates in anti-cancer drug-induced apoptosis in human cervical cancer HeLa cells. They also show that the synergistic effects of TRAIL in combination with either ER stressors or genotoxic anti-cancer drugs are nearly exclusively mediated via an increased activation of Bid-induced apoptosis signalling.

## Introduction

The members of the Bcl-2 family of proteins are key players in cellular life and death decisions during apoptosis [1]. The two pro-apoptotic, multi-domain Bcl-2 family proteins Bax and Bak control mitochondrial outer membrane permeabilization (MOMP) during apoptosis, an important process leading to the activation of caspase-dependent and caspase-independent cell death pathways [2]. Indeed, Bax and Bak have been considered to represent the ‘gateway’ of apoptosis, as cells from Bax/Bak double knock-out animals are resistant to most apoptosis-inducing stimuli [3], [4]. The activation of Bax and Bak involves conformational changes, membrane insertion, and oligomerisation, with their pro-apoptotic activity inhibited by anti-apoptotic, multidomain Bcl-2 family proteins such as Bcl-2 and Bcl-xL.

Bid is a member of the Bcl-2-homology domain (BH)-3-only family of pro-apoptotic proteins that initiate apoptotic cell death [5]. Similar to the BH3-only proteins Puma and Bim, it is able to neutralize the anti-apoptotic activity of most anti-apoptotic Bcl-2 family members, including Bcl-2, Bcl-xL, Mcl-1, and Bcl-w [6]–[8]. There is also strong evidence supporting the hypothesis that Bid is able to directly activate Bax and Bak to trigger the release of caspase-activating factors from mitochondria [9]–[11]. Bid is also involved in mitochondrial remodelling during apoptosis and maybe required for the complete release of caspase-activating factors from mitochondria [12]. *bid* is a p53 target gene [13]. However, Bid is constitutively expressed in many transformed and non-transformed cells, and its main mechanism of activation involves a series of posttranslational modifications including proteolytic cleavage, myristoylation, phosphorylation, and translocation to mitochondria [14]–[17]. During death receptor-induced apoptosis, Bid is activated through cleavage by caspase-8 or caspase-10, generating a truncated form, t-Bid, [18], [19]. Bid has also been reported to be proteolytically activated by other proteases, including caspase-3, linking Bid to feed-back processes during apoptosome-dependent apoptosis [20], and caspase-2, linking Bid to other stress-induced apoptosis pathways [21], [22]. Bid has also been suggested to be cleaved by granzymes, calpains, and cathepsins in the loop connecting alpha helices 2 and 3, generating cleavage products that may also induce the release of caspase-activating factors from mitochondria [23]–[29]. Finally, full-length Bid may also have the capacity to translocate to mitochondria and activate apoptosis without previous cleavage by caspase-8 or other proteases [30]–[32]. Taken together these data suggest that Bid may be a central sensor linking stress signaling and (patho)physiological protease activation to the activation of the mitochondrial apoptosis pathway.

Interestingly, *bid*-deficient mice presented with a relatively mild phenotype, but showed a prominent resistance to Fas-dependent apoptosis of hepatocytes [33]. Recently, Bid has also been suggested to play a role in anti cancer drug- and endoplasmic reticulum (ER) stress-induced apoptosis in human cells [34]–[36], although recent studies in *bid*-deficient murine cells could not identify such a role for Bid [37]. The present study was therefore undertaken to comprehensively explore the role of Bid in death receptor-, genotoxic drug-, and ER stress-induced apoptosis in human cervical cancer HeLa cells.

## Results

### Generation of *bid*-deficient HeLa Cells

**Figure 1:**
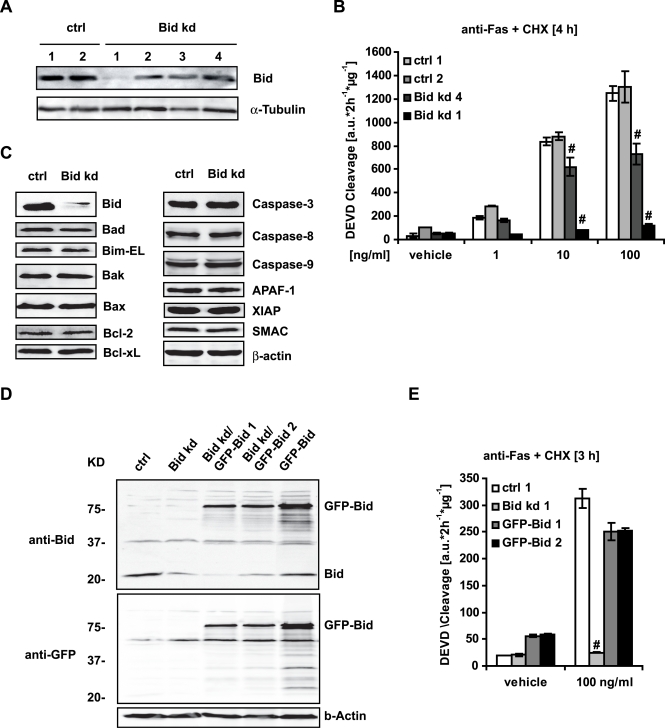
Generation of stable Bid deficient HeLa cells. A) Cells were stably transfected with plasmids encoding one of three different *bid*-specific shRNAs or a scrambled control shRNA as described in Materials and Methods. For analysis of the *bid* knockdown, cells were subjected to Western blotting with a polyclonal Bid antibody. α-Tubulin served as a loading control. B) Control cells expressing the scrambled (Ctrl 1, Ctrl 2), or the *bid* specific shRNA (Bid kd 4) and clone (Bid kd 1) were incubated with Cycloheximide (CHX) (1 µg/ml) and an agonistic Fas antibody or vehicle (clone CH11) at the indicated concentrations or vehicle for 4 h. Caspase-3 like activity was measured by cleavage of the fluorigenic substrate Ac-DEVD-AMC. Data are means+/−SD from n = 3 separate experiments. # p<0.05: difference from control cells (Ctrl). C) Cell lysates were subjected to Western blotting with antibodies for the indicated proteins, as described in Materials and Methods. D) HeLa Bid kd cells were stably transfected with a vector coding for YFP-*bid*-CFP (GFP-Bid); cell lysates were subjected to Western blotting with GFP and Bid antibodies. E) HeLa Bid kd cells expressing GFP-Bid were treated with CHX (1 µg/ml) and an activating Fas antibody (100 ng/ml). Controls were treated with vehicle for 4 h; caspase-3 like activity was measured by cleavage of the fluorogenic substrate Ac-DEVD-AMC. Data are means+/−SD from n = 3 separate experiments. # p<0.05 difference from control cells (Ctrl).

In order to study the role of Bid in death receptor- and drug-induced apoptosis, gene silencing using three different shRNA constructs targeting different regions of *bid* was performed as described in Materials and Methods. Western blot analysis of established stable cell lines demonstrated different levels of reduction in Bid protein expression compared to cells transfected with a scrambled control sequence (Fig. 1 A). These reduced Bid levels correlated with reduced executioner caspase activity in response to an activating Fas antibody (clone CH11), as determined by analyzing the cleavage of the fluorogenic caspase substrate Ac-DEVD-AMC (Fig. 1 B). The Bid knockdown clone 1 (hereafter designated as HeLa Bid kd) was further analyzed as this clone demonstrated maximal knockdown of Bid expression, whereas the expression of other BH3-only proteins, Bcl-2 family members, or other key proteins involved in caspase activation were not altered (Fig. 1 C). Furthermore, re-introduction of ectopically expressed *bid* (Fig. 1 D) was able to re-sensitize this clone to Fas-induced caspase activation and apoptosis (Fig. 1 E), indicating that the reduction in caspase activity after death receptor activation was due to the lack of Bid expression.

### Bid is required for Fas/CD95- and TRAIL-induced cell death of HeLa Cells

**Figure 2:**
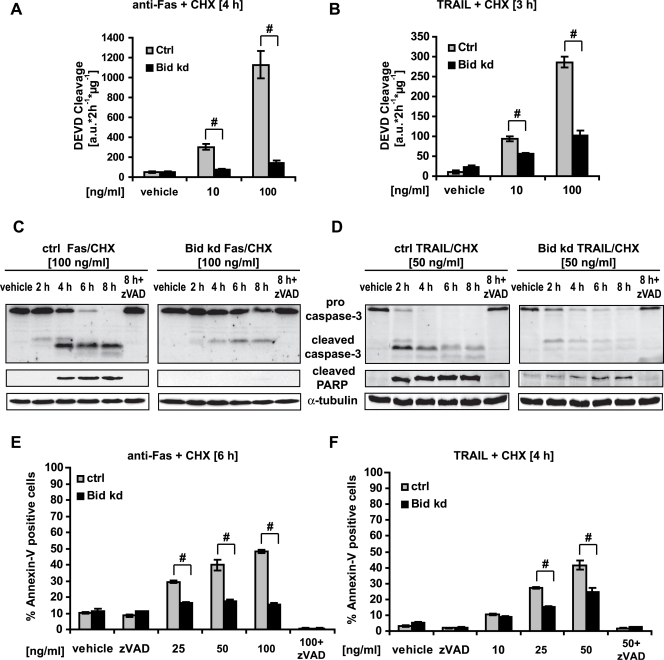
Bid depletion abrogates the death receptor-mediated apoptotic pathway. Control and HeLa Bid kd were pre-incubated with the pan-caspase inhibitor zVAD-fmk (100 µM) for 1 h followed by treatment for the specified times with CHX (1 µg/ml) in combination with an agonistic Fas antibody or recombinant TRAIL at the indicated concentrations. Controls were treated with vehicle. A, B) Caspase-3 like activity was measured by cleavage of the fluorogenic substrate Ac-DEVD-AMC. Data are means+/−SD from n = 3 separate experiments. # p<0.05 difference from control cells (Ctrl). C, D) Cells were treated as indicated and lysates were subjected to Western blotting with a polyclonal caspase-3, a monoclonal Poly-ADP-Ribose Polymerase (PARP) antibody and a monoclonal α-tubulin antibody. E, F) Cells were treated as indicated and apoptosis was assessed by flow cytometric evaluation of Annexin-V FITC conjugated binding to phosphatidylserine in non-permeabilized cells. Data are means+/−SD from n = 3 separate experiments. # p<0.05 difference from control cells (Ctrl).

**Figure 3:**
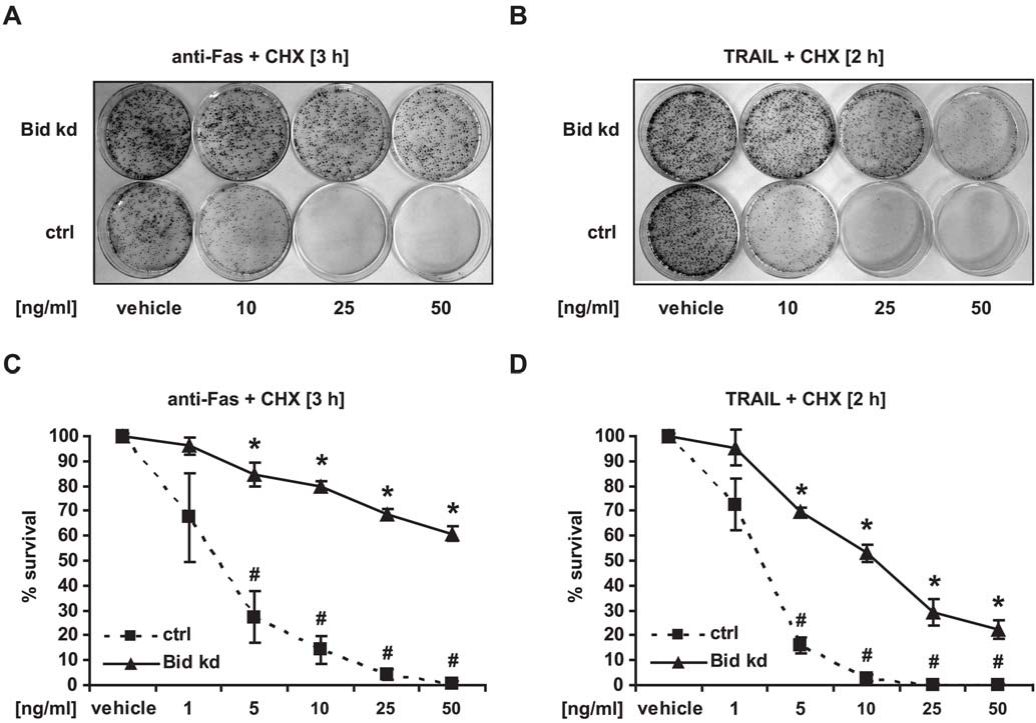
Inhibition of death receptor-induced apoptosis correlates with enhanced clonogenic survival in HeLa Bid kd cells. Control cells and HeLa Bid kd cells were treated with CHX (1 µg/ml) in combination with an agonistic FAS antibody or recombinant TRAIL for the indicated times. Controls were treated with vehicle. After incubation, 1000 cells were transferred to 60 mm dishes and cultured with fresh medium for 14 days. Then colonies were fixed, stained with methylene blue and counted. A, B) Colony formation in representative dishes is shown. C, D) Graphical representation of the percentage of colonies after treatment compared to control cells treated with vehicle (100%). Data are means+/−SD from at least two independent experiments performed in triplicate. # p<0.05: difference from control cells (Ctrl). ∗ p<0.05: difference from Bid kd cells treated with vehicle.

We next compared the effect of the *bid* knockdown to death receptor-induced apoptosis triggered by the activating Fas antibody or by treatment with recombinant human TRAIL (TR). Dose-response analyses demonstrated that the knockdown of *bid* potently reduced DEVD cleavage activity in response to both stimuli compared to a control clone transfected with a scrambled sequence (Fig. 2 A, B). Procaspase-3 processing, poly-ADP-ribose polimerase (PARP) cleavage and phosphatidylserine exposure were analyzed by Western blotting and flow cytometry (Fig. 2 C, D, E, F). The kinetics of procaspase-3 processing corresponded with the generation of the caspase-cleaved 85 kDa PARP-fragment, the latter being detectable 2 h after TRAIL/cycloheximide (Chx) and 4 h after Fas/Chx incubation. In HeLa Bid kd cells, procaspase-3 processing, cleaved PARP levels, and the accumulation of Annexin V-positive cells were significantly reduced. Importantly, the knockdown of *bid* also significantly increased the clonogenic survival of HeLa cells in response to TRAIL and Fas activation as analyzed in colony formation assays (Fig. 3 A, B, C, D).

**Figure 4:**
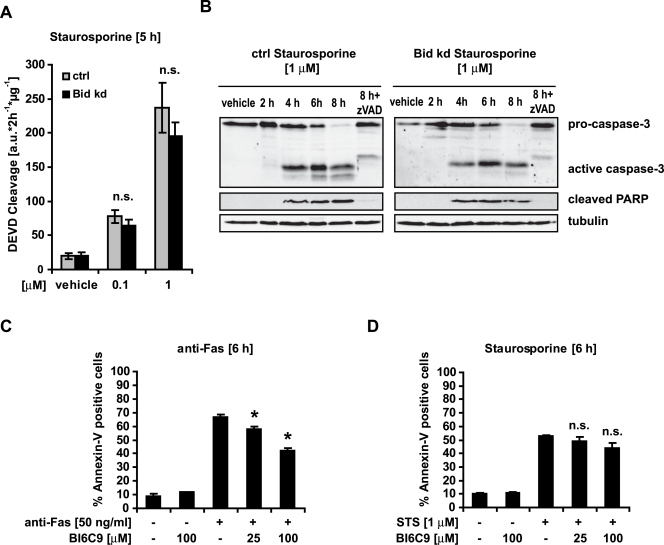
Bid is dispensable for apoptosis triggered by the kinase inhibitor Staurosporine. Control and HeLa Bid kd cells were pre-incubated with the pan-caspase inhibitor zVAD-fmk (100 µM) for 1 h before treatment with the indicated concentrations of Staurosporine for the indicated times. A) Caspase-3 like activity was measured by cleavage of the fluorogenic substrate Ac-DEVD-AMC. Data are means+/−SD from n = 3 separate experiments. n.s. = not significant versus control (Ctrl). B) Cells were treated as indicated and lysates were subjected to Western blotting with a polyclonal caspase-3, a monoclonal Poly-ADP-Ribose Polymerase (PARP) antibody and a monoclonal α-tubulin antibody. C, D) The small molecule Bid inhibitor BI6C9 mimics the effects of the knockdown of Bid in HeLa cells. Parental HeLa cells were pre-incubated with the indicated concentrations of BI6C9 for 16 h prior to treatment with an agonistic Fas antibody, Staurosporine (STS) or vehicle, for 6 h. Apoptosis was assessed by flow cytometric evaluation of Annexin-V FITC conjugated binding to phosphatidylserine in non-permeabilized cells. Data are means+/−SD from n = 3 separate experiments. ∗ p<0.05: difference from control cultures treated with Fas antibody or Staurosporine in the absence of BI6C9.

In contrast to death receptor-induced apoptosis, HeLa Bid kd cells were not protected from caspase activation and cell death after exposure to the protein kinase inhibitor Staurosporine (STS), demonstrating that these cells did not acquire a general resistance against apoptosis activation (Fig. 4 A, B).

To confirm the results of the *bid* knockdown in a different approach, HeLa cells were treated with the synthetic Bid inhibitor BI6C9 [38]. Cells treated with BI6C9 were significantly protected against death receptor-induced apoptosis (Fig. 4 C), while the inhibitor had no significant effect on cell death induced by STS (Fig. 4 D).

### ER stress-induced apoptosis by Tunicamycin does not require Bid

**Figure 5:**
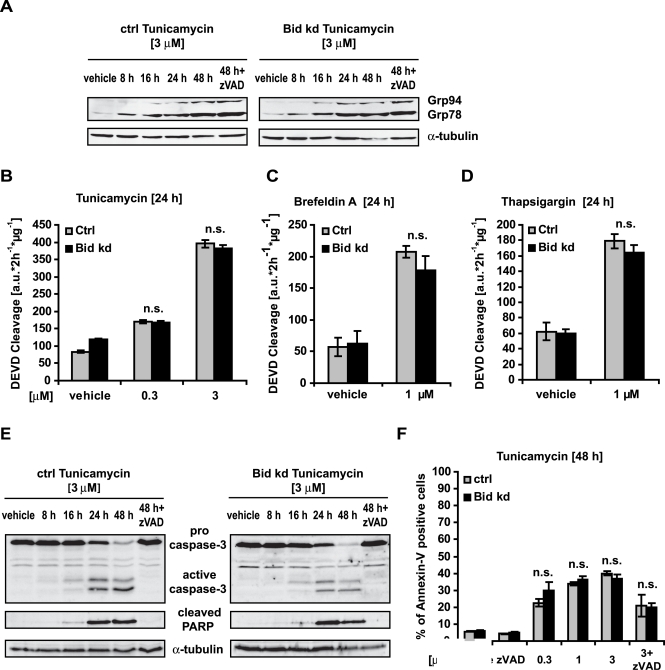
Bid is dispensable for endoplasmic reticulum (ER) stress-induced apoptosis. Control cells and HeLa Bid kd cells were pre-incubated with the pan-caspase inhibitor zVAD-fmk (100 µM) where specified for 1 h before treatment with the ER stressors Tunicamycin, Brefeldin A, and Thapsigargin at the indicated concentrations for the indicated times. A) Cell lysates were subjected to Western blotting with a monoclonal KDEL antibody, which detects Grp94 and Grp78, and a monoclonal α-tubulin antibody. B, C, D) Caspase-3 like activity was measured by cleavage of the fluorigenic substrate Ac-DEVD-AMC. Data are means+/−SD from n = 3 separate experiments. n.s. = not significant versus control (Ctrl). E) Cells were treated as indicated and lysates were subjected to Western blotting with a polyclonal caspase-3 antibody, a monoclonal Poly-ADP-Ribose Polymerase (PARP) antibody and a monoclonal α-tubulin antibody. F) Apoptosis was assessed by flow cytometric evaluation of Annexin-V FITC conjugated binding to phosphatidylserine in non-permeabilized cells. Data are means+/−SD from n = 3 separate experiments. n.s. = not significant versus control (Ctrl).

It has been previously suggested that ER stress can trigger caspase-8 activation and downstream induction of the intrinsic apoptosis pathway via Bid processing [34], [39]. To test this hypothesis in the HeLa Bid kd cells, ER stress was induced using Tunicamycin (Tu), Thapsigargin (Th) and Brefeldin A. The antibiotic Tunicamycin inhibits *N*-linked glycosylation of nascent proteins, leading to an accumulation of hypoglycosylated proteins in the ER lumen, resulting in the activation of ER stress responses and, if persisting, to ER stress-induced apoptosis. Treatment of HeLa Bid kd cells and control HeLa cells with Tunicamycin resulted in a similar induction in protein levels of the ER resident molecular chaperones Grp78 and Grp94, which are known ER stress response target genes (Fig. 5 A), but also resulted in similar levels of caspase activation (Fig. 5 B, E), apoptosis and cell death (Fig. 5 F). These results suggested that Bid did not affect the unfolded protein response (UPR) and was not required for ER stress-induced apoptosis. Similar results were obtained when ER stress was induced by Brefeldin A or Thapsigargin, which inhibit ER to Golgi transport and the sarco/endoplasmic reticulum Ca^2+^ ATPases, respectively (Fig. 5 C, D).

### The knockdown of *bid* abolishes the synergistic effect of Tunicamycin and TRAIL on apoptosis in HeLa cells

**Figure 6:**
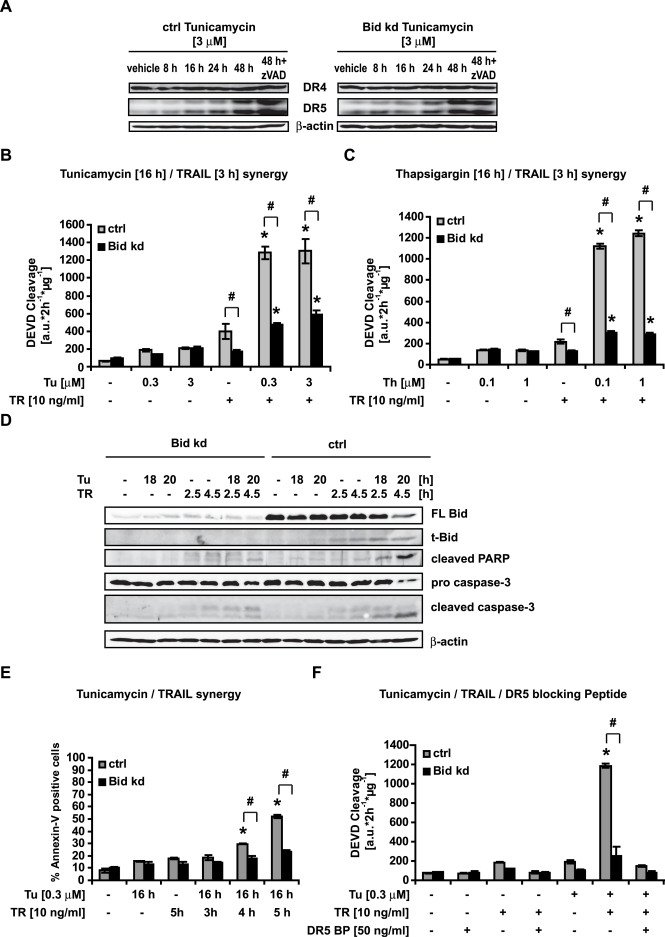
The synergistic effects of ER Stress and TRAIL on apoptosis depend on Bid. A) HeLa Control and HeLa Bid kd cells were pre-incubated with the pan-caspase inhibitor zVAD-fmk for 1 h where specified; cells were subsequently treated with Tunicamycin or vehicle for the indicated times. Cell lysates were subjected to Western blotting with a polyclonal DR4, a polyclonal DR5, and a monoclonal β-actin antibody. B, C) Control cells and HeLa Bid kd cells were pre-incubated for 16 h with the indicated concentrations of Tunicamycin, Thapsigargin or vehicle before treatment with TRAIL (10 ng/ml). Caspase-3 like activity was measured by cleavage of the fluorogenic substrate Ac-DEVD-AMC. Data are means+/−SD from n = 3 separate experiments. ∗ p<0.05: difference from Tunicamycin, Thapsigargin or TRAIL individual treatments. # p<0.05: difference from control cells (Ctrl). D) Control cells and HeLa Bid kd cells were pre-incubated with Tunicamycin (0.3 µM) or vehicle for the indicated times followed by treatment with TRAIL (10 ng/ml) for the indicated times. Cell lysates were subjected to Western blotting with a polyclonal Bid, a polyclonal caspase-3, a polyclonal Poly-ADP-Ribose Polymerase (PARP) antibody and a monoclonal α-tubulin antibody. E) Control cells and HeLa Bid kd cells were pre-incubated with Tunicamycin (0.3 µM) for 16 h followed by treatment with recombinant TRAIL (10 ng/ml) for the indicated times. Apoptosis was assessed by flow cytometric evaluation of Annexin-V FITC conjugated binding to phosphatidylserine in non-permeabilized cells. Data are means+/−SD from n = 3 separate experiments. ∗ p<0.05: difference from Tunicamycin or TRAIL individual treatments. # p<0.05: difference from control cells (Ctrl). F) Control cells and HeLa Bid kd cells were pre-incubated with a DR5 blocking peptide (50 ng/ml) for 1 h followed by pre-treatment with Tunicamycin (0.3 µM) or vehicle for 16 h. Subsequently, cells were treated with recombinant TRAIL for 3 h. Caspase-3 like activity was measured by cleavage of the fluorogenic substrate Ac-DEVD-AMC. Data are means+/−SD from n = 3 separate experiments. ∗ p<0.05: difference from Tunicamycin or TRAIL individual treatments. # p<0.05: difference from control cells (Ctrl).

ER stress induced by Brefeldin A has been shown previously to synergize with an agonistic Fas antibody [40], and Tunicamycin has been shown to synergize with TRAIL-induced apoptosis by increasing the expression of the TRAIL receptor death receptor 5 (DR5) [41]. Although our results indicate that Bid is not directly involved in ER stress-mediated apoptosis, it may play an important role in the synergistic effects of ER stress-inducing agents and death receptor ligands. Treatment of HeLa control cells or Bid kd cells with Tunicamycin potently up regulated DR5, both at the mRNA (data not shown) and the protein level, while death receptor 4 (DR4) levels were not affected (Fig. 6 A). Pre-treatment with low doses of the ER stressors Tunicamycin and Thapsigargin followed by exposure to low doses of TRAIL induced a dramatic increase in caspase activity to levels higher than predicted from the individual treatments (Fig. 6 B, C). These synergistic effects were confirmed by Western blotting which detected increased procaspase-3 processing, cleavage of PARP, and cleavage of Bid (Fig. 6 D). In addition, flow cytometry analysis of Annexin-V binding indicated enhanced phosphatidylserine exposure after combined treatment with TRAIL and Tunicamycin (Fig. 6 E). The knockdown of *bid* completely abolished these synergistic effects between TRAIL and Tunicamycin (Fig. 6 B, C, D, E). Furthermore, a DR5 blocking peptide (DR5 BP) also abolished the synergistic effect between Tunicamycin and TRAIL (Fig. 6 F), demonstrating that signalling through DR5 and, downstream from this through Bid, was required for this synergistic effect.

### Bid deficiency protects against Etoposide-, Doxorubicin- and Oxaliplatin induced apoptosis

**Figure 7:**
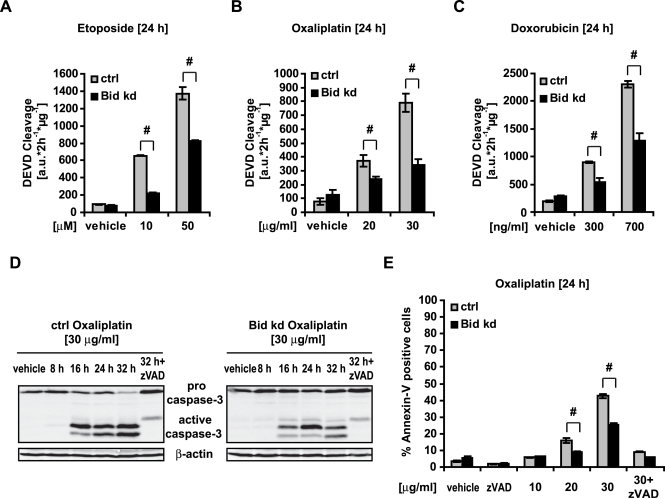
Bid participates in apoptosis induced by genotoxic drugs. HeLa control and HeLa Bid kd cells were treated with the indicated concentrations of Etoposide, Oxaliplatin and Doxorubicin, or vehicle, for 24 h; cells were pre-incubated with the pan-caspase inhibitor zVAD-fmk (100 µM) for 1 h where specified. A, B, C) Caspase-3 like activity was measured by cleavage of the fluorogenic substrate Ac-DEVD-AMC. Data are means+/−SD from n = 3 separate experiments. # p<0.05 difference from control cells (Ctrl). D) Cells were treated as indicated and lysates were subjected to Western blotting with a polyclonal Bid, a polyclonal caspase-3, a polyclonal Poly-ADP-Ribose Polymerase (PARP) antibody and a monoclonal α-tubulin antibody. E) Apoptosis was assessed by flow cytometric evaluation of Annexin-V FITC conjugated binding to phosphatidylserine in non-permeabilized cells. Data are means+/−SD from n = 3 separate experiments. # p<0.05 difference from control cells (Ctrl).

**Figure 8:**
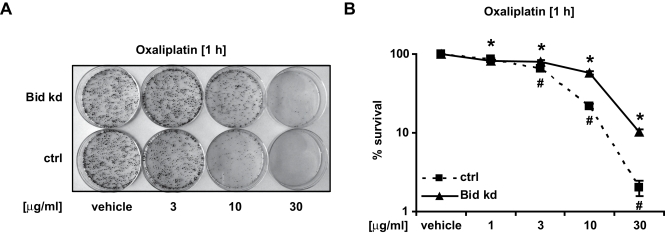
Knockdown of Bid improves clonogenic survival after treatment with Oxaliplatin. HeLa Control and HeLa Bid kd cells were treated with the indicated concentrations of Oxaliplatin (1 h) or vehicle. After incubation, 1000 cells were transferred to 60 mm dishes and cultured in fresh medium for 14 days. Then colonies were fixed, stained with methylene blue and counted. A) Colony formation in representative dishes is shown. B) Graphical representation of the percentage of colonies after treatment compared to control cells treated with vehicle (100%). Data are means+/−SD from at least two independent experiments performed in triplicate. # p<0.05: difference from control cells (Ctrl). ∗ p<0.05: difference from Bid kd cells treated with vehicle.

**Figure 9:**
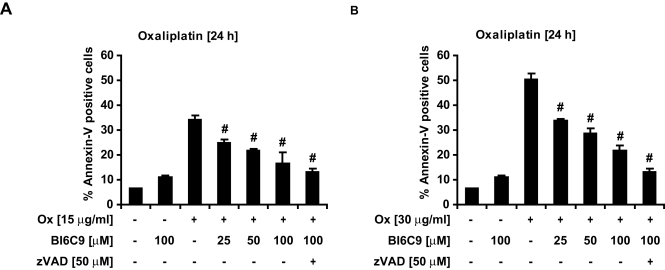
The synthetic Bid inhibitor BI6C9 protects HeLa cells from apoptosis induced by genotoxic drugs. A, B) Parental HeLa cells were pre-incubated with the indicated concentrations of BI6C9 or the pan-caspase inhibitor z-VAD (100 µM) for 16 h, prior to treatment with 15 µg/ml (A) or 30 µg/ml (B) Oxaliplatin or vehicle for 24 h. Apoptosis was assessed by flow cytometric evaluation of Annexin-V FITC conjugated binding to phosphatidylserine in non-permeabilized cells. Data are means+/−SD from n = 3 separate experiments. # p<0.05 difference from control cultures treated with Oxaliplatin in the absence of BI6C9.

To assess the potential contribution of Bid to DNA damage-induced apoptosis in HeLa cells, we treated HeLa Bid kd cells and HeLa control cells with the anticancer drugs Etoposide (Eto), Oxaliplatin (Ox), and Doxorubicin. Detection of effector caspase activation by activity assays indicated that the knockdown of *bid* was able to confer a partial resistance to genotoxic drugs (Fig. 7 A–C). A more detailed analysis was performed with the anti-cancer drug oxaliplatin. Western blotting, flow cytometry analysis of Annexin-V binding (Fig. 7 D, E) and clonogenic survival assays (Fig. 8 A, B) indicated that the knockdown of *bid* conferred a partial resistance to oxaliplatin. These protective effects of the *bid* knockdown were confirmed by treatment of HeLa cells with the Bid inhibitor BI6C9 (Fig. 9 A, B).

**Figure 10:**
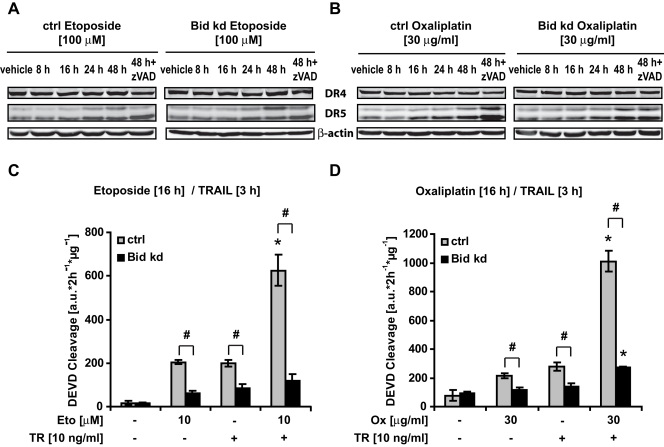
Bid is necessary for the synergistic activity between genotoxic drugs and TRAIL. A, B) HeLa Control and HeLa Bid kd cells were treated with Etoposide (10 µM) or Oxaliplatin (30 µg/ml) for the indicated times; the pan-caspase inhibitor zVAD (100 µM) was added to the cells 1 h prior to treatment where specified; cell lysates were subjected to Western blotting with a polyclonal DR4, a polyclonal DR5, and a monoclonal β-actin antibody. C, D) HeLa control and HeLa Bid kd cells were pre-incubated with Etoposide (10 µM), Oxaliplatin (30 µg/ml), or vehicle for 16 h followed by treatment with TRAIL (10 ng/ml) for 3 h. Caspase-3 like activity was measured by cleavage of the fluorogenic substrate Ac-DEVD-AMC. Data are means+/−SD from n = 3 separate experiments. ∗ p<0.05: difference from Etoposide, Oxaliplatin or TRAIL individual treatments. # p<0.05 difference from control cells (Ctrl).

We next investigated the role of Bid in the potential synergism between genotoxic drugs and TRAIL. Oxaliplatin and Etoposide both upregulated DR5 receptor expression in a time-dependent manner, while DR4 levels remained constant (Fig. 10 A, B). Similar to our data obtained with ER stressors, we observed potent synergistic effects of Oxaliplatin and Etoposide with TRAIL which was completely abolished in Bid-deficient cells (Fig. 10 C, D).

### Additive effects of Bid and PUMA in mediating Oxaliplatin-induced apoptosis

**Figure 11:**
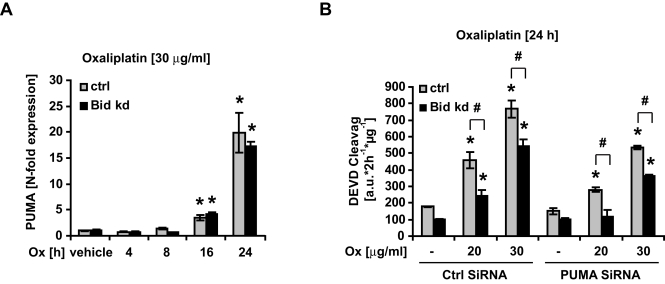
Additive effects of the BH3-only proteins PUMA and Bid during genotoxic stress-induced apoptosis. A) HeLa Control and HeLa Bid kd cells were treated with Oxaliplatin (30 µg/ml); *puma* mRNA levels were evaluated by qPCR at the indicated time points. Data are means+/−SD from n = 3 separate experiments. n.s. = not significant versus control (Ctrl). B) After transient transfection with scrambled siRNA or *puma* siRNA, HeLa control and HeLa Bid kd cells were treated with the indicated concentrations of Oxaliplatin for 24 h. Caspase-3 like activity was measured by cleavage of the fluorogenic substrate Ac-DEVD-AMC. Data are means+/−SD from n = 3 separate experiments. ∗ p<0.05: difference from vehicle treated control cultures transfected with control siRNA (Ctrl SiRNA). # p<0.05 difference between respective HeLa control and Bid kd cells.

Since the BH3-only gene *puma* has been shown to be a pivotal regulator of apoptosis induced by DNA-damaging anticancer drugs in many types of cancer cells and in particular to mediate Oxaliplatin-induced apoptosis [42], we finally determined the relative contributions of Bid and PUMA in apoptosis induced by oxaliplatin. We compared the effects of Oxaliplatin on *puma* gene expression in HeLa control cells and HeLa Bid kd cells. *puma* mRNA levels were potently induced by Oxaliplatin as evaluated by qPCR (Fig. 11 A). We could not observe significant differences in *puma* mRNA upregulation between HeLa control and HeLa Bid kd cells (Fig. 11 A), indicating that the stress signaling pathways mediating *puma* expression were similarly activated in control and Bid kd cells, and that the induction of *puma* occurred independently of Bid. Transient RNA interference against *puma* was able to reduce *puma* mRNA expression by 47.58+/−8.7% and 64.61+/−3.2% after 24 h and by 59.71+/−1.1% and 69.67+/−0.1% after 48 h in the HeLa control and HeLa Bid kd cells, respectively (n = 3 experiments; normalized to *β-actin* mRNA expression and compared to cultures transfected with a scrambled siRNA sequence). Treatment with *puma* siRNA significantly reduced levels of apoptosis in HeLa control cells exposed to Oxaliplatin (Fig. 11 B). The knockdown of *puma* led to a further decrease of apoptosis in the HeLa Bid kd cells, indicating that both BH3-only proteins cooperate in mediating apoptosis triggered by Oxaliplatin.

## Discussion

Truncated Bid (t-Bid) can be generated by the activity of caspase-8 and -10 [15], [43] and has been described to be a very potent BH3-only protein [6]–[8], [9]–[11], [44]–[46]. Furthermore, Bid is constitutively expressed in many tumours and is cleaved by several stress-activated proteases other than caspase-8 or -10 [21], [23]–[29]. Given these special characteristics of Bid compared to other BH3-only proteins, we established a stable knockdown of *Bid* expression by a gene silencing approach in HeLa cervical carcinoma cells and addressed the functional importance of Bid expression in death receptor- and stress-induced apoptosis in a human cancer cell line. In line with the well established role of Bid in linking the extrinsic and intrinsic pathways of apoptosis, depletion of Bid by stable RNA interference lead to a pronounced cellular resistance to Fas/CD95- and TRAIL-mediated caspase activation and apoptosis. In further experiments, we could confirm this finding using the synthetic, cell-permeable Bid inhibitor BI6C9. Finally, silencing of *Bid* also significantly increased the clonogenic survival of HeLa cells treated with either the Fas/CD95 activating antibody or recombinant TRAIL. These results indicate that in HeLa cervical cancer cells, apoptosis signaling through Fas/CD95 and TRAIL occurs almost exclusively in a Bid dependent manner, with the direct activation of caspase 3 by caspase-8 insufficient to compensate for a loss of Bid expression and to induce cell death. Importantly, these results also suggest that in HeLa cervical cancer cells, other, potentially MOMP-independent cell death pathways [47] appear to play only a minor role in response to Fas/CD95 activation or TRAIL.

Activation of caspase-8 has also been suggested to play a significant role during ER stress-induced apoptosis [34], [48], [49]. To test the hypothesis that the caspase-8/Bid pathway is also a central mediator of ER stress-induced cell death in human cancer cells, we treated HeLa control cells and HeLa Bid kd cells with apoptosis-inducing concentrations of three ER stressors, each having a different mechanism of action (Tunicamycin, Thapsigargin, and Brefeldin A). However, inhibition of Bid by RNA interference (Fig. 6) or by treatment with BI6C9 (authors' unpublished data) did not reduce caspase activation and cell death induced by these three ER stress stimuli. Our findings therefore support the data from a previous study of Borner and co-workers [50] where Bid expression was not required for ER stress mediated cell death. However, cells were dramatically protected from apoptosis when subtoxic concentrations of the ER stressors Tunicamycin or Thapsigargin were combined with subtoxic concentrations of TRAIL. Indeed, the synergistic effect of Tunicamycin and TRAIL was completely abolished by the Bid knockdown. In subsequent experiments, we were able to show that ER stress induced by Tunicamycin or Thapsigargin was associated with enhanced expression of the death receptor DR5 as reported previously by our and other groups [51]–[53], while we did not detect any expression changes of the death receptor DR4. The synergistic effects of TRAIL and Tunicamycin could be completely blocked with a specific DR5 blocking peptide demonstrating that signaling through DR5 was required for this synergism [52], [53]. However, the relevance of upregulated DR5 expression for enhanced TRAIL DISC formation and stress-triggered sensitization of cancer cells to TRAIL-induced apoptosis is controversially discussed [41], [54], [55], as it may also result from an enhanced binding of caspase-8 to the DISC. Nevertheless, our data indicate that in either scenario, signaling through Bid is absolutely required for the synergism of ER stressors and TRAIL.

Despite the considerable interest in TRAIL or agonistic TRAIL-receptor antibodies as anticancer agents, accumulating evidence suggests that indeed mono-therapies with these agents may have limited success, but that synergistic treatments with other apoptosis inducers may be a good rationale to efficiently activate apoptosis in many types of cancers [53], [56]–[59]. Indeed a similar synergism, described previously in other tumor cells [60]–[63], and its strict dependence on Bid expression were observed when TRAIL was combined with the genotoxic drugs, Oxaliplatin or Etoposide. It is well established that many anti-cancer drugs including Oxaliplatin and Etoposide induce the tumor suppressor p53 which is able to transcriptionally activate *DR5* expression but also *bid* expression [13], [64]. These endogenous cell death ‘back up’ mechanisms, in co-operation with an enhanced ability to recruit caspase-8 to the DISC, therefore provide a biological framework that can be therapeutically explored by these combined treatment paradigms. Interestingly, in our previous trancriptome profiling of cells undergoing ER stress mediated apoptosis [51] we noted a distinct lack of p53 target genes, suggesting that the upregulation of DR5 by ER stressors may occur independent of p53 expression. Therefore, in tumours where the p53 signaling pathway is lost/mutated the TRAIL agonists in combination with ER stressors may be of better therapeutic benefit than their utilization in combination with genotoxic drugs.

In cancer cells treated with genotoxic drugs only, the p53 target gene *puma* has been suggested to largely mediate the pro-apoptotic activity of p53 [65]–[67]. In our study, *puma* was potently activated by Oxaliplatin in HeLa control and Bid kd cells, and transient RNA interference against *puma* was able to significantly reduce apoptosis in both cell lines. Despite the prominent role of PUMA in mediating genototoxic drug-induced apoptosis, several lines of evidence support the notion that Bid also partially contributes to apoptosis induced by DNA-damage [68]–[71]. In contrast to its death-inducing role in apoptosis, Bid has also been implicated in *protecting* cells from genotoxic stress by promoting cell cycle arrest, thus facilitating DNA repair and potentially cellular survival [70], [72]. Our Bid-deficient cells displayed significantly reduced levels of caspase activation in response to the DNA-damaging anti-cancer drugs Etoposide, Oxaliplatin and Doxorubicin. Follow-on experiments with Oxaliplatin demonstrated reduced cell death as well as an increased clonogenic survival in the Bid-deficient cells, but this effect was less dramatic than that observed after treatment with the death ligands TRAIL or Fas. Apart from the involvement of alternative BH3 only proteins, in particular PUMA in Oxaliplatin-induced apoptosis, the proposed dual function of Bid in apoptosis and preservation of genomic integrity may also explain the less pronounced effect of the Bid knockdown on cell death and clonogenic survival in response to Oxaliplatin. Our results differ from a previous report in which the effect of *bid* gene deletion was explored in non-transformed murine cells [37]. In cancer cells, Bid's proapoptotic function may predominate over its proposed protective function. The integration of BH3 only proteins into stress-induced signalling pathways may have also evolved differently between murine and human cells [73], [74].

A further level of complexity in regard to the role of Bid in the response to genotoxic stress lies in the fact that Bid can be activated through several distinct mechanisms after DNA damage. In addition to its transcriptional induction, Bid has been shown to be proteolytically activated by the initiator caspase, caspase-2, and generation of t-Bid has been suggested to be essential for apoptosis induced by caspase-2 [21]. Activation of calpains has also been implicated in this context [75]. Furthermore, in some scenarios, full length Bid is also able to translocate to mitochondria and trigger the intrinsic pathway of apoptosis without previous proteolytic activation [14], [30]–[32], [77]–[78]. Bid therefore appears to be a target of several signaling pathways that co-operate with the induction of other BH3 Only Proteins such as PUMA in mediating genotoxic drug-induced apoptosis.

Collectively, our data demonstrate that Bid is an indispensable component of death receptor-induced cell death, but also participates in DNA damage-induced apoptosis of human cervical cancer HeLa cells. Importantly, our data also show that the synergistic effects of the death ligand TRAIL in combination with either ER stressors or DNA damaging anti-cancer drugs are nearly exclusively mediated via an increased activation of Bid-induced apoptosis signaling.

## Materials and Methods

### Materials

Human recombinant TRAIL was purchased from Leinco Technologies (Universal Biologicals, Gloucestershire, UK). Caspase substrate N-acteyl-Asp-Glu-Val-Asp-7-amino-4-methyl-coumarin (Ac-DEVD-AMC) and the pan-caspase inhibitor Z-Val-Ala-Asp(O-methyl)-fluoromethylketone (zVAD-fmk) were obtained from Bachem (St. Helen's, UK). All other chemicals came in analytical grade purity from Alexis (Blessington, Ireland) or Sigma-Aldrich (Dublin, Ireland).

### Cell culture

HeLa cells were grown in RPMI 1640 medium supplemented with 10% (v/v) heat-inactivated fetal calf serum, 2 mM glutamine, 100 U/ml penicillin, and 100 mg/ml streptomycin (Sigma-Aldrich) in a humidified 5% CO_2_ containing atmosphere at 37°C. Cells were kept in logarithmic growth phase by routinely passaging them twice a week and were plated 24 h prior to treatments.

### Generation of stable bid knockdown and control clones

The following shRNA sequences specific for human Bid mRNA were designed using the Dharmacon siRNA design tool (http://www.dharmacon.com/sidesign/): Bid-1 sense (5′-AAGCTGTTCTGACAACAGC-3′), Bid-2 sense (5′-AAGGAGAAGACCATGCTGG-3′) and Bid-3 sense (5′-AAGAATAGAGGCAGATTCT-3′). Bid-specific or control shRNA duplexes were ligated into the p*Silencer* 2.1-U6 hygro vector (Ambion, Cambridgeshire, UK) via their *Bam*H I and *Hin*d III sites. To generate stable knock down cell lines HeLa cells were transfected with the different shRNA constructs using Metafectene (Biontex, Munich, Germany) according to the manufacturer's instructions. 24 h post transfection the cells were (serially) diluted, transferred to 96-well plates and stable clones were selected using hygromycin B (160 µg/ml).

Stable clones expressing ectopically re-introduced Bid were generated as outlined above by using a pFRET-Bid plasmid (kind gift from Dr. R. Onuki, National Institute of Advanced Industrial Science and Technology, Tsukuba, Japan) [79], expressing a YFP-Bid-CFP fusion protein.

### Transient RNA interference

siRNA duplexes targeting *puma* mRNA were designed utilizing the RNA workbench (www.rnaworkbench.com). The annealed *puma* (5′-GAUGGCCCAGCCUGUAAGAUACUdTdT-3′) and control siRNA (5′-UUCUCCGAACGUGUCACGUdTdT-3′) duplexes were purchased from Sigma Proligo (Paris, France). 24 h after seeding into six-well plates, cells were transfected with 100 nM of the siRNA duplex using Metafectene Pro (Biontex, Munich, Germany) as per manufacturer's instructions. 24 h post transfection cells were treated as indicated.

### SDS-PAGE and Western blotting

Preparation of cell lysates and western blotting was performed as previously described [80]. The following primary antibodies were used: a rabbit polyclonal APAF-1 (Chemicon, Carrigtwohill, Ireland), a rabbit polyclonal Bad or caspase-3 (Cell Signaling, Bray, Ireland), a mouse monoclonal Bak or Bcl-2 (Santa Cruz, Heidelberg, Germany), a rabbit polyclonal Bax (Upstate, Carrigtwohill, Ireland), a rabbit polyclonal Bcl-X (BD Biosciences, Erembodgem, Belgium), a mouse monoclonal Bim or caspase-8 (Alexis Biochemicals, Blessington, Ireland), a goat polyclonal Bid (R&D Systems, Abingdon, UK), a rabbit polyclonal caspase-9 (Calbiochem, Darmstadt, Germany), a rabbit polyclonal DR4 or DR5 (Abcam, Cambridge, UK), a rabbit poyclonal GFP (Clontech, Oxford, UK), a mouse monoclonal KDEL (Stressgen, York, UK), a rabbit polyclonal cleaved PARP (NEB, Bray, Ireland), a rabbit polyclonal smac/DIABLO (R&D Systems), a mouse monoclonal XIAP (BD Biosciences), a mouse monoclonal β-actin or α-tubulin (Sigma Aldrich, Dublin, Ireland). Horseradish peroxidase-conjugated secondary antibodies (Jackson Immuno Research, Cambridge, UK) were detected using SuperSignal West Pico Chemiluminescent Substrate (Pierce) and imaged using a FujiFilm LAS-3000 imaging system (Fuji).

### Flow cytometry

The measurement Annexin-V-FITC-staining of apoptotic cells was performed on a Cyflow ML16 flow cytometer (Partec, Münster, Germany) as described previously [81].

### Real-time qPCR

Total RNA was extracted using the RNeasy Mini kit (Qiagen, Hilden, Germany) and 2 µg per sample were reverse-transcribed into first strand cDNA using random hexamer primers (50 pmol) and MMLV Reverse Transcriptase (Invitrogen, Dun Laoghaire, Ireland). Quantitative real-time PCR was conducted using the QuantiTech SYBR Green PCR kit (Qiagen) and the LightCycler (Roche Diagnostics, Lewes, UK) as described previously [81]. Primers were designed using Primer3 software (http://frodo.wi.mit.edu/cgi-bin/primer3/primer3_www.cgi) and came from Sigma-Genosys (Cambidge, UK). Sense and antisense primers were: *β-actin*: TCACCCACACTGTGCCCATCTA and CAGCGGAACCGCTCATTGCCAA; *puma*: CCATCTCAGGAAAGGCTGTT and ACGTTTGGCTCATTTGCTCT.

### Determination of caspase-3-like protease activity

Preparation of cell lysates to determine their caspase-3-like protease-activity was performed as previously described [80]. Cleavage of the fluorigenic substrate DEVD-AMC was monitored by measuring the accumulation of fluorescent AMC after 1 and 2 h using a GENios microplate reader (Tecan, Crailsheim, Germany). Protein content was determined using the Coomassie Plus Protein assay reagent (Pierce, Dublin, Ireland). Caspase activity was expressed as change in fluorescence per µg of protein and hour.

### Colony formation assay

Cells were treated as indicated before transferring 1,000 cells to 60 mm dishes and culturing them for two weeks to allow for colony formation. Then the medium was removed, colonies were fixed and stained with a solution containing ethanol (50%) and methylene-blue (0.25%) (Sigma-Aldrich, Dublin, Ireland) for 45 min and the number of colonies per plate was counted.

### Statistics

Data are given as means±SD or SEM. For statistical comparison, t-test or one-way ANOVA followed by Tukey test were employed using SPSS software (SPSS GmbH Software, Munich, Germany). P-values smaller than 0.05 were considered to be statistically significant.
